# ICG-liver test versus new biomarkers as prognostic markers for prolonged length of stay in critically ill patients - a prospective study of accuracy for prediction of length of stay in the ICU

**DOI:** 10.1186/s13613-014-0019-7

**Published:** 2014-07-08

**Authors:** Bernhard Zoller, Katharina Spanaus, Rahel Gerster, Mario Fasshauer, Paul A Stehberger, Stephanie Klinzing, Athanasios Vergopoulos, Arnold von Eckardstein, Markus Béchir

**Affiliations:** 1 Surgical Intensive Care Medicine, University Hospital of Zurich, Raemistrasse 100, Zurich, CH-8091, Switzerland; 2 Institute of Clinical Chemistry, University and University Hospital Zurich, Zurich, Switzerland; 3 Swiss Paraplegic Center, Nottwil, Switzerland

**Keywords:** Copeptin, MR-proANP, pro-ADM, ICG-Liver test, ICG-PDR, pLOS, Length of stay in the ICU

## Abstract

**Background:**

Prognostic abilities of medical parameters, which are scoring systems, measurements and biomarkers, are important for stratifying critically ill patients. Indocyanine green plasma disappearance (ICG-PDR) is an established clinical tool for the assessment of liver perfusion and function. Copeptin, MR-proANP and pro-ADM are biomarkers whose prognostic value is still unclear. The goal of this prospective study was to evaluate ICG-PDR, copeptin, MR-proANP and pro-ADM to predict prolonged length of stay (pLOS) in the ICU.

**Methods:**

This study was conducted as a prospective single center study including 110 consecutively admitted ICU patients. Primary endpoint was prolonged length of stay (pLOS) in the ICU, defined as more than three days of stay there.

**Results:**

ROC analysis showed an AUC of 0.73 for ICG-PDR, 0.70 for SAPS II, 0.65 for MR-proANP, 0.64 for pro-ADM and 0.54 for copeptin for pLOS in the ICU.

**Conclusions:**

The prediction of pLOS in the ICU might be better by means of ICG-PDR than with the new biomarkers copeptin, MR-proANP or pro-ADM. Nevertheless, there is more need for research to evaluate whether ICG-PDR is an overall prognostic marker for pLOS.

**Trial registration:**

(ClinicalTrials.gov number, NCT01126554).

## Background

The prognostic abilities of medical parameters, which are scoring systems, measurements and biomarkers, are important for stratifying critically ill patients [[1]] to optimize resources and further investigation in this area is warranted.

The Simplified Acute Physiology Score (SAPS II) is a validated scoring system to predict mortality in critically ill patients [[2]]. Regardless of the underlying diagnosis, the combination of 17 variables allows reliable outcome prediction. SAPS II models have proved to be competitive to the SOFA (Sequential Organ Failure Assessment) based score models [[3]]. However, SAPS II scoring is less reliable for prediction of length of stay (LOS) in the ICU [[4],[5]].

Indocyanine green plasma disappearance rate (ICG-PDR) is closely correlated to hepatic function due to its hepatic metabolization. The non-invasive ICG measurement, based on pulse dye measurement using a finger-clip, has been shown to correlate well with the classical measurements [[6]] and is non-invasive and easy to use. ICG-PDR has proven to predict mortality in critically ill patients comparable to the SAPS II score [[7]], as well as to predict complications in liver cirrhosis patients [[8]] and posthepatectomy liver failure [[9]]. The capability of ICG-PDR to predict LOS in the ICU remains elusive.

Several new biomarkers are under investigation in the ICU for critically ill patients.

Copeptin, a peptide of 39 amino acids, is the C-terminal part of pro-arginine vasopressin also called C-terminal-pro-arginine vasopressin (CT-proAVP). It reveals several physiologic actions: arteriolar vasoconstriction (V1-receptor mediated), an anti-diuretic effect in the kidneys (V2-receptor mediated) [[10]] and is probably involved in the secretion of ACTH (V3-receptor mediated) [[11]]. It has been proven to predict mortality in patients with community acquired pneumonia [[12]], stroke [[13]], and traumatic brain injury [[14]].

Atrial natriuretic peptide (ANP) is a counter-regulatory hormone synthesized in the heart, being cleaved and released during hemodynamic stress or myocardial injury. High mid-region proANP levels (MR-proANP) are associated with high mortality in patients with acute myocardial infarction [[15]] and chronic obstructive pulmonary disease (COPD) exacerbation [[16]].

Proadrenomedullin (pro-ADM) is a 185 amino acid-long precursor of the vasodilator adrenomedullin (52 amino acids). It is released during severe infection, especially septic shock and is influenced by age, body mass index and glomerular filtration rate in healthy volunteers [[17]]. Like MR-proANP, it has been proven to predict mortality in patients with COPD exacerbation [[18]] and acute myocardial infarction [[19],[20]].

While these biomarkers have proven useful to predict mortality in different settings, to our knowledge no data are available concerning their predictive value of ICU pLOS. The goal of this study was to investigate the accuracy of ICG-PDR, SAPS II, copeptin, MR-proANP, pro-ADM and other established biomarkers for prediction of pLOS in the ICU.

## Methods

### Study design

This prospective single center study was conducted at the surgical ICU (10 beds, 920 patients in the year 2010) at the University Hospital of Zurich from 1 August 2010 to 31 October 2010. During this period, all patients admitted to our ICU were eligible for inclusion regardless of underlying disease or reason for admission. Exclusion criteria were: age below 18 years, pregnancy, inclusion in another study, patients or relatives not able to understand the German language or known iodine allergy (a component of ICG). The aim was the comparison of the prognostic value of the ICG-liver test and SAPS II score on the one hand with new biomarkers (copeptin, pro-ADM and MR-proANP), and on the other hand to established biomarkers (bilirubin, aspartate aminotransferase (AST), alanine aminotransferase (ALT), alkaline phosphatase (AP), International Normalized Ratio (INR), hemoglobin, white blood cell (WBC) count, activated partial thromboplastin time (aPTT), dDimer, cystatin C, C-reactive protein (CRP), procalcitonin (PCT), IL-6, the B-type natriuretic peptide (NT-proBNP) Primary endpoint was prolonged length of stay (pLOS) in the ICU, represented by more than three days. A power calculation was performed with the assumption of 5% mortality and 30% of pLOS in the patient population; therefore 110 patients with an estimated drop-out of 10% were prospectively included. Initially, mortality was considered as primary endpoint but the financial burden of such a large sample size (hundreds of patients) was unaffordable. Therefore, the study was performed with the endpoint pLOS.

The study was approved by the local ethical committee and informed consent was achieved by all patients or next of kin. The trial was registered as ClinicalTrials.gov number, NCT 01126554.

### Study protocol

After achievement of informed consent, patients were included into the study. Baseline characteristics were collected. Reason for ICU admission was classified as visceral surgery, thoracic surgery, medical disease and others. Blood samples used for the analysis were drawn within two hours after admission. The ICG-liver testing was performed non-invasively within 60 minutes after ICU admission by pulse spectrophotometry (LiMON®, Pulsion Medical Systems AG, Munich, Germany). Following an intravenous bolus injection of ICG (0.25 mg/kg; ICG Pulsion Medical Systems AG, Munich, Germany), plasma ICG concentrations were determined by pulse spectrophotometry with a finger-clip sensor. Using two near-infrared wavelengths, the plasma disappearance rate of ICG (ICG-PDR) was calculated automatically by the time course of the blood ICG concentration. Normal values are defined as ICG-PDR ≥ 16%/minute. SAPS II score was calculated after 24 hours.

### Laboratory analysis

Venous whole blood samples were collected in tubes containing either ethylenediaminetetraacetic acid (EDTA) or lithium heparin. Copeptin, MR-proANP and pro-ADM were determined in EDTA plasma by Time-Resolved Amplified Cryptate Emission (TRACE) technology on the KRYPTOR analyzer (BRAHMS, Henningsdorf, Germany).

Plasma levels of PCT and NT-proBNP were measured by electrochemiluminescence immunoassays on a COBAS 8000 modular analyzer (Roche Diagnostics, Rotkreuz, Switzerland). IL-6 levels were analyzed using a chemiluminescence enzyme immunoassay on an Immulite 2500 analyzer (Siemens Healthcare Diagnostics, Zurich, Switzerland) and cystatin C was measured by immuno-nephelometry on a Behring nephelometer system (Dade Behring, Dudingen, Switzerland).

Plasma concentrations of AST, ALT, AP, bilirubin and CRP, were measured on a COBAS 8000 modular analyzer (Roche Diagnostics, Rotkreuz, Switzerland).

### Statistical analysis

All subjects were grouped into the two clusters: ICU stay ≤ three days and prolonged ICU stay (>three days). Univariate statistics of the biomarkers and tests applied in this study were done by Mann-Whitney *U*-test or Fishers exact test as appropriate. All *P*-values were considered two-sided and statistical significance was considered as *P* < 0.05. Biomarkers and tests showing statistical significance were further examined by receiver operating characteristics (ROC) analysis. Comparison of ROC analysis was done by comparison of area under the curve (AUC).

Accuracy of ICG, SAPS II, IL-6, dDimer, NT-proBNP, MR-proANP and pro-ADM were tested and the cut offs defined (significance and specificity based) by ROC analysis. Statistics were done with SPSS for Windows version 18.0 (SPSS, Chicago, IL, USA).

## Results

### Baseline characteristics

We screened 117 patients admitted to the ICU during the study period. Seven patients were not enrolled due to exclusion criteria. After a drop-out of four patients, because of two lost blood sample sets and two withdrawals of consent, the data of 106 patients were analyzed.

Forty-one of the patients were female (38.7%) and 65 male (61.3%). ICU mortality was 2.8% (n = 3), whereas hospital mortality was 6.6% (n = 7). The four diagnostic groups were: visceral surgery 53 patients (50.0%), thoracic surgery 27 patients (25.5%), medical group 7 patients (6.6%) and others 19 patients (17.9%). Thirty-eight patients were ventilated on admission to the ICU, whereas 28 could be extubated within the first 12 hours after admission. In the visceral surgery group, 20 underwent liver surgery (wedge resection, left or right hemihepatectomy). Eight patients had preexisting liver dysfunction (2 Child C and 6 Child B cirrhosis).

Overall, 25 (23.6%) patients had a prolonged length of stay in the ICU. There was no statistical significant difference between the diagnostic groups in respect to pLOS or normal LOS. The median of LOS was 4.2 days in all patients.

### Univariate data

The univariate analysis of laboratory values between the short stay and prolonged stay groups in the ICU and baseline criteria are shown in Table 1. There was no statistical difference in baseline criteria between the diagnostic groups. There was a statistical significance between the two groups concerning ICG-PDR (*P* = 0.03), bilirubin (*P* = 0.003), AST (*P* = 0.02), ALT (*P* = 0.05), SAPS II (*P* = 0.01), IL-6 (*P* = 0.005), Ddimer (*P* = 0.02), aPTT (0.05), MR-proANP (*P* = 0.03) and pro-ADM (*P* = 0.03) while copeptin did not show a statistical significant difference (*P* = 0.67).

**Table 1:** Baseline, clinical and laboratory parameters

**Parameter**	**ICU stay > 3 days**	**ICU stay ≤ 3 days**	***P***
	**(n = 25)**	**(n = 81)**	
Age (years)	64 (61 to 70)	65 (48 to 73)	0.89
ICG-PDR (%/minute)	17 (12 to 24)	22.5 (17 to 29)	0.03
Bilirubin (μmol/l)	15 (9 to 31)	9 (6 to 15)	0.003
AST (U/l)	84 (35 to 439)	36 (23 to 90)	0.02
ALT(U/l)	57 (23 to 403)	29 (16 to 70)	0.05
AP (U/l)	63 (46 to 132)	52 (41 to 75)	0.26
Quick (%)	86 (46 to 106)	89 (63 to 105)	0.50
Hemoglobin (g/dl)	9 (8 to 13)	10 (9 to 12)	0.79
WBC (g/l)	10 (9 to 12)	10 (7 to 14)	0.82
aPTT (seconds)	33 (29 to 40)	29 (25 to 33)	0.05
dDimer (mg/l)	3 (2 to 5)	2 (1 to 3)	0.02
Cystatin C (mg/l)	1 (1 to 1)	1 (1 to 1)	0.72
CRP (mg/l)	31 (5 to 81)	15 (5 to 70)	0.87
PCT (μg/l)	1 (1 to 2)	2 (1 to 4)	0.41
IL-6 (ng/l)	403 (190 to 853)	177 (55 to 314)	0.005
NT-proBNP (ng/l)	745 (111 to 1875)	245 (68 to 957)	0.09
MR-proANP (pmol/l)	268 (119 to 342)	140 (94 to 214)	0.03
Pro-ADM (nmol/l)	2 (1 to 3)	1 (1 to 2)	0.03
Copeptin (pmol/l)	96 (20 to 171)	94 (28 to 230)	0.67
SAPS II (points)	31 (26 to 43)	24 (18 to 30)	0.01

Data presented as median (IQR). AP, alkaline phosphatase; aPTT, activated partial thromboplastin time; ALT, alanine aminotransferase; AST, aspartate aminotransferase; CRP, C-reactive protein; ICG-PDR, indocyanine green plasma disappearance rate; IL, interleukin; MR-proANP, pro-atrial natriuretic peptide; NT-proBNP, B-type natriuretic peptide; PCT, procalcitonin; Pro-ADM, proadrenomedullin; SAPS II, Simplified Acute Physiology Score; WBC, white blood cells. Quick, prothrombin time.

### ROC analysis

The ROC analysis is shown in Table 2. ICG-PDR (AUC 0.73) and SAPS II (AUC 0.7) did perform better for prediction for prolonged length of stay in the ICU than the new biomarkers MR-proANP (AUC 0.65), pro-ADM (AUC 0.64) and copeptin (AUC 0.54). There was no statistical difference between ICG-PDR and SAPS II (*P* = 0.78).

**Table 2:** **Receiver operating characteristics** (**ROC) statistics**

**Parameter**	**AUC (95% CI)**	**Cut-off**	**Sensitivity (%)**	**Specificity (%)**
ICG (%)	0.73 (0.56 to 0.88)	11.5	71	71
SAPS II (points)	0.70 (0.54 to 0.84)	25.5	76	64
IL-6 (ng/l)	0.68 (0.52 to 0.84)	289	59	74
dDimer (mg/l)	0.67 (0.49 to 0.85)	2.5	59	72
NT-proBNP (ng/l)	0.67 (0.51 to 0.84)	947	53	82
MR-proANP (pmol/l)	0.65 (0.49 to 0.81)	209	59	74
Pro-ADM (nmol/l)	0.64 (0.48 to 0.81)	2.5	41	89

ICG, indocyanine green; IL, interleukin; MR-proANP, pro-atrial natriuretic peptide; NT-proBNP, B-type natriuretic peptide; Pro-ADM, proadrenomedullin; SAPS II, Simplified Acute Physiology Score.

## Discussion

The main finding of this study is the value of ICG-PDR in prediction of pLOS in the ICU. The measurement is easy to perform and, in contrast to the SAPS II score, consists of only one value and not of seventeen values, which made its application time-saving and quick. As ICG-PDR reflects liver function, this finding confirms that impaired liver function is of crucial importance in the assessment and prognosis of critical care patients. Impaired liver function shows a wide spectrum of immunosuppressant effects, metabolic and pharmacological disturbances and coagulation disorders. Thus, ICG-PDR might be a representative surrogate for a summation of these effects with negative influence for intensive care patients. This suggestion is supported by the fact that in our study the patients with pLOS also had higher serum bilirubin, AST and ALT levels as well as a prolonged aPTT. There was no better discrimination of MR-proANP and pro-ADM between long- and short-term LOS in the ICU than ICG-PDR. A potential explanation might be that the heterogeneous population presented with impaired liver function at a grade of severity, but the MR-proANP and pro-ADM pathways were activated by different underlying pathologies and probably do not reflect the severity of disease. Nevertheless, there are patient populations in which those markers showed a good predictive accuracy in prediction of outcome [[18],[21],[22]], but still we lack data about ICU patients. A recent study by Guignant *et al*. reported an association between elevated pro-ADM levels and nosocomial infection in patients after septic shock, whereas copeptin and MR-proANP showed no correlation in this population [[23]].

Copeptin failed to discriminate LOS between long-term and short-term patients; therefore, its prognostic value seems to be weak in this area. One reason might be that the main trigger for copeptin release - as a part of the vasopressin pathway - is hypovolemia, which, in our study population of postsurgical and resuscitated medical ICU patients, was definitely not the case. In our institution, most of the unstable patients are monitored by the PiCCO-system (Pulsion, Munich, Germany), Swan-Ganz catheter or by means of echocardiography, which might have led to an adequate volemia at the point of admission.

One important study limitation is the heterogeneous and small patient population, which might explain some of the results. Nevertheless, our cohort was a representative ICU population of a university hospital center with patients of considerable pathological complexity and severity. Furthermore, we assessed only one data point of every marker per patient on admission. Whether a repetitive regimen of measurement at, for instance, day 3 or 5, would have shown different results, remains unclear. These findings might help - but only in combination with clinical signs and the experience of the ICU staff - to utilize and optimize the scarce ICU resources by early stratification, which might lead to earlier but safe discharge to intermediate care units.

## Conclusion

The prediction of pLOS in the ICU might be better by means of ICG-PDR than with the new biomarkers copeptin, MR-proANP or pro-ADM. Nevertheless, there is a need for further research to evaluate whether ICG-PDR is an overall prognostic marker for pLOS.

## Abbreviations

ACTH: adrenocorticotropic hormone

ALT: alanine transaminase

ANP: atrial natriuretic peptide

AP: alkaline phosphatase

aPTT: activated partial thromboplastin time

AST: aspartate transaminase

AUC: area under the curve

COPD: chronic obstructive pulmonary disease

CRP: C-reactive protein

CT-proAVP: C-terminal-pro-arginine vasopressin

EDTA: ethylenediaminetetraacetic acid

ICG-PDR: indocyanine green plasma disappearance rate

IL: interleukin

INR: International Normalized Ratio

LOS: length of stay

MR-proANP: pro-atrial natriuretic peptide

NT-proBNP: B-type natriuretic peptide

PCT: procalcitonin

pLOS: prolonged length of stay

pro-ADM: proadrenomedullin

ROC: receiver operating characteristics

SAPS II: Simplified Acute Physiology Score

SOFA: Sequential Organ Failure Assessment

TRACE: Time-Resolved Amplified Cryptate Emission

WBC: white blood count

## Competing interests

The authors declare that they have no competing interests.

## Authors’ contributions

Each author have participated sufficiently in the work to take public responsibility for appropriate portions of the content: MF, BZ and RG collected the majority of the data and drafted parts of the manuscript. KS, AV performed laboratory tests and drafted parts of the manuscript. PAS performed statistical analysis and drafted parts of the manuscript. SK redrafted the article and did most of the revisions. AV and MB led the project, collected parts of the data, performed additional statistical analysis and drafted parts of the manuscript. All authors read and approved the final manuscript.
